# Associations of Mental Health and Personal Preventive Measure Compliance With Exposure to COVID-19 Information During Work Resumption Following the COVID-19 Outbreak in China: Cross-Sectional Survey Study

**DOI:** 10.2196/22596

**Published:** 2020-10-08

**Authors:** Yihang Pan, Meiqi Xin, Changhua Zhang, Willa Dong, Yuan Fang, Wenhui Wu, Mingzhe Li, Jun Pang, Zilong Zheng, Zixin Wang, Jinqiu Yuan, Yulong He

**Affiliations:** 1 Precision Medicine Center Scientific Research Center, The Seventh Affiliated Hospital Sun Yat-sen University Shenzhen China; 2 Big Data Center The Seventh Affiliated Hospital Sun Yat-sen University Shenzhen China; 3 JC School of Public Health and Primary Care The Chinese University of Hong Kong Hong Kong; 4 Center for Digestive Disease The Seventh Affiliated Hospital Sun Yat-sen University Shenzhen China; 5 Department of Health Behavior Gillings School of Global Public Health University of North Carolina at Chapel Hill Chapel Hill, NC United States; 6 Department of Early Childhood Education The Education University of Hong Kong Hong Kong; 7 Center for Urology The Seventh Affiliated Hospital Sun Yat-sen University Shenzhen China; 8 Clinical Research Center The Seventh Affiliated Hospital Sun Yat-sen University Shenzhen China

**Keywords:** COVID-19, information exposure, risk, communication, mental health, personal preventive measures, China, cross-sectional, public health, prevention

## Abstract

**Background:**

Risk and crisis communication plays an essential role in public health emergency responses. The COVID-19 pandemic has triggered spontaneous and intensive media attention, which has affected people’s adoption of personal preventive measures and their mental health.

**Objective:**

The aim of this study was to investigate the associations between exposure to COVID-19–specific information and mental health (depression and sleep quality) and self-reported compliance with personal preventive measures (face mask wearing and hand sanitizing). We also tested whether these associations were moderated by thoughtful consideration of the veracity of the information to which people were exposed.

**Methods:**

A cross-sectional, closed web-based survey was conducted among a sample of 3035 factory workers at the beginning of work resumption following the COVID-19 outbreak in Shenzhen, China. A stratified two-stage cluster sampling design was used for recruitment. Multivariate linear and logistic regression models were used for the analyses.

**Results:**

The prevalence of probable moderate-to-severe depression was 170/3035 (5.6%), while that of good or excellent sleep quality was 2110/3035 (69.5%). The prevalence of self-reported consistent face mask wearing in public places was 2903/3035 (95.7%), while that of sanitizing hands every time after returning from public spaces or touching public installations was 2151/3035 (70.9%). Of the 3035 respondents, 1013 to 1638 (33.3% to 54.0%) reported >1 hour of daily exposure to COVID-19–specific information through web-based media and television. After controlling for significant background variables, higher information exposure via television and via newspapers and magazines was associated with better sleep quality and higher compliance with hand sanitizing. Higher exposure via unofficial web-based media was associated with higher compliance with hand sanitizing but was also associated with higher depressive symptoms. In contrast, higher exposure through face-to-face communication was associated with higher depressive symptoms, worse sleep quality, and lower compliance with hand sanitizing. Exposure to information about positive outcomes for patients with COVID-19, development of vaccines and effective treatments, and heroic stories about frontline health care workers were associated with both better mental health and higher compliance with preventive measures. Higher overall information exposure was associated with higher depressive symptoms among participants who were less likely to carefully consider the veracity of the information to which they were exposed; it was also associated with better sleep quality among people who reported more thoughtful consideration of information veracity.

**Conclusions:**

This study provides empirical evidence of how the amount, sources, and contents of information to which people were exposed influenced their mental health and compliance with personal preventive measures at the initial phase of work resumption in China. Thoughtful consideration of information quality was found to play an important moderating role. Our findings may inform strategic risk communication by government and public health authorities during the COVID-19 pandemic.

## Introduction

Risk and crisis communication plays an essential role in public health emergency responses [1]. During public health emergencies, the media is an essential tool used by government and public health authorities to manage crises [2], and the public relies on media to understand the situation [3]. Infectious disease pandemics, such as severe acute respiratory syndrome (SARS) and Middle East respiratory syndrome (MERS) trigger spontaneous and intensive media attention, which could have effects on various public responses [4,5]. One of the most desirable public responses is the adoption of personal preventive measures. Universal face mask wearing [6] and hand hygiene [7] are strongly advocated by the World Health Organization (WHO) and have been widely implemented during the COVID-19 pandemic [8,9]. Media exposure has been demonstrated to increase health knowledge among the public, which in turn encourages desirable preventive behaviors during infectious disease pandemics [4,10].

In addition, people facing a public health emergency often experience a range of negative emotions. During the COVID-19 outbreak, the unknown cause of the disease, fatal outcomes, and interruption of daily routines likely increased levels of negative emotions [11]. The COVID-19 pandemic also triggered mental health problems among the general public, such as stress, panic, depression, and anxiety [12-14]. The media tend to overemphasize risk and sensationalize crises, and repeated information exposure through the media may result in public panic and fear. According to Social Amplification Theory, this panic and fear can be further amplified through the exchange of information on diverse media platforms or through face-to-face communication [15]. Mental distress provoked by information exposure can fuel the proliferation of misinformation and amplify adverse health outcomes and maladaptive responses [16-18].

The importance of web-based media during public health emergencies has been increasing over time. Individuals have turned to web-based media during pandemics to find information related to safety precautions, news updates, and protective equipment. For example, during the SARS and MERS outbreaks, Chinese and South Korean people turned to the internet to find information that was unavailable on traditional media channels [19,20]. During the early phase of the COVID-19 outbreak in 2020, 94% of Hong Kong residents considered social media to be their most important information source [21]. Compared to traditional media, web-based media platforms (such as social media) provide not only information but also emotional expression. It has been observed that negative emotions are more likely to be conveyed on social media than positive emotions during an infectious disease pandemic [22]. Another study suggested that social media has had a significant impact on the spread of fear and panic during the COVID-19 outbreak [23]. Studies conducted during the MERS outbreak of 2015 indicated that increased exposure to MERS-specific information through social media was simultaneously associated with higher adoption of personal preventive measures and higher levels of negative emotions [4,24]. However, traditional media consumption (eg, television and newspapers) did not influence personal preventive measures or mental health [4,24].

Previous studies have shown that people are likely to react emotionally rather than rationally during a disease outbreak [25]. For example, in one study [26], participants were less able to pay attention to external information and did not trust authorities. Moreover, they were more likely to absorb negative rather than positive information. Although web-based media is a powerful tool for disseminating information, there are concerns related to inaccurate data, unverified rumors, and even malicious misinformation on these platforms [4]. With the COVID-19 outbreak, a global epidemic of misinformation has also been spreading rapidly through social media, which poses a serious additional challenge for public health efforts [27,28]. In response, the WHO and many other public health authorities are verifying rumors and providing evidence-based information [29,30]. In this information environment, it is also important to understand how audiences process the obtained information (eg, the extent of thoughtful consideration of the veracity of health information) and whether these processes will affect their behaviors and mental health during the pandemic.

This study targeted workers who resumed work in China at the beginning of work resumption. Although the implementation of strict nationwide control measures (eg, closure of all unessential businesses) effectively controlled the pandemic, it severely damaged China’s economy [31]. Therefore, the Chinese government scaled up work resumption starting on February 20, 2020 [32]. However, there were concerns that the increase in public contact after work resumption would result in a second wave of COVID-19 in China [33]. Implementing effective risk communication can help workers comply with personal preventive measures without amplifying panic or negative emotions, which is crucial to achieve a balance of disease control with revitalization of the economy.

To the best of our knowledge, there have been no studies investigating behavioral or mental health outcomes of exposure to COVID-19–specific information at the initial phase of work resumption following the COVID-19 outbreak. The objective of this study was to investigate the associations between COVID-19–specific information exposure and four outcome variables, including depression, sleep quality, self-reported consistent face mask wearing, and hand hygiene. The study also tested whether the associations between information exposure and outcomes varied as a function of thoughtful consideration of the veracity of information to which the participants were exposed.

## Methods

### Settings and Participants

**Figure 1:**
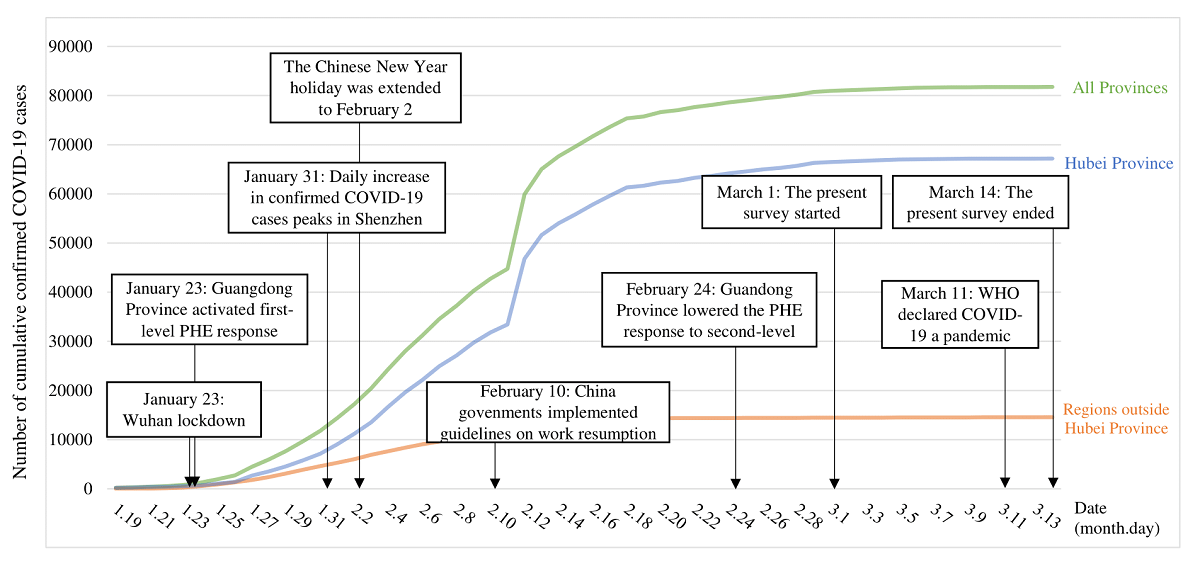
Background of the present survey, including the trend of cumulative confirmed COVID-19 cases in mainland China and critical responses to COVID-19 in Shenzhen, a city in Guangdong Province. PHE: public health emergency; WHO: World Health Organization.

A cross-sectional, closed web-based survey was conducted from March 1 to 14, 2020, in Shenzhen, China. Approximately 65.1% of the residents of this city are internal migrants, and 34.3% are factory workers [34]. During the COVID-19 pandemic, Shenzhen has been one of the most severely affected regions in China outside of Hubei Province, which is the epicenter of the pandemic. The daily increase in confirmed COVID-19 cases peaked at 60 on January 31. By March 1, 2020, 100 factories in Shenzhen had resumed work. A stratified two-stage cluster sampling approach was used for recruitment. First, 14 factories were randomly selected by the research team. Of these 14 factories, 10 (71%) manufactured electronic devices, 2 (14%) manufactured watches, 1 (7%) manufactured beverages, and 1 (7%) manufactured biotechnology products. All full-time employees aged ≥18 years who had resumed work in these factories were then invited to complete a web-based survey. Details of the study background are shown in Figure 1.

### Procedure

We developed a web-based questionnaire using Questionnaire Star, a commonly used web-based survey platform in China. The link to access the web-based questionnaire was shared in WeChat, a popular messaging app. In addition to national guidelines, the Shenzhen government requested that each factory establish WeChat groups including all employees as part of the preparation for work resumption. A designated coordinator responsible for COVID-19 control in each factory facilitated the data collection. This coordinator posted the study information and the link to access the web-based questionnaire in the WeChat group and invited all eligible workers who had resumed work to participate. The coordinator also sent out reminders in the WeChat groups twice per week during the study period. These designated coordinators did not participate in the survey. The coordinators and participants were asked not to disseminate the survey access link to people outside their factories. Before starting the web-based survey, the participants read a statement indicating that participation was voluntary, refusal to participate would have no effect on them, the survey would not collect personal contacts or identifying information, and the data would be kept strictly confidential and would only be used for research purposes. Web-based informed consent was sought. Each individual WeChat account was allowed to access the web-based survey once. The web-based survey platform performed completeness checks before each questionnaire was submitted. Participants were able to review and change their responses using a Back button. Upon completion of the survey, an electronic coupon of ¥10 (US $1.30) was sent to each participant. All data were stored in the server of the survey platform and were protected by a password. Only the corresponding authors had access to the database. Of 4158 workers who had resumed work in these factories on March 1, 2020, 3035 completed the web-based survey. The overall response rate was 73.0%. Ethics approval was obtained from the Seventh Affiliated Hospital, Sun Yat-sen University (reference: KY-2020-005-001).

### Measures

#### Background Variables

Sociodemographic data such as age, gender, highest education level, marital status, monthly personal income, status as frontline workers or management staff, and type of factories the respondents were working in were collected.

#### COVID-19–Specific Information Exposure

Five items assessed daily average time (hours) of COVID-19–specific information exposure through different sources from January 24 (the first day of the Chinese New Year holiday) through the survey date. Sources of health information included official web-based media (media accounts of government institutions), unofficial web-based media (media accounts of private organizations or self-published accounts), television, newspapers and magazines, and face-to-face communication. The Overall Information Exposure Scale was constructed by summing the individual item scores (1=almost none, 2=<1 hour, 3=1-2 hours, 4=3-4 hours, and 5=>4 hours). A higher score on the scale indicated exposure to a higher amount of COVID-19–specific information.

Participants were also asked to report their frequencies of exposure to information on the eight most popular topics related to COVID-19 during the outbreak (0=almost never to 3=always). These topics included statistics about the COVID-19 pandemic (number of confirmed or suspected cases, deaths, and recoveries), positive information about governmental responses to COVID-19 (eg, building mobile clinics, mobilizing national resources to epidemic areas), negative information about governmental responses to COVID-19 (eg, insufficient supply of protective equipment by some local governmental organizations), information about the development of vaccines and effective treatments, heroic stories about frontline health care workers, positive information about patients with COVID-19 (eg, recovery, discharge from hospital), negative information about patients with COVID-19 (eg, severe symptoms and deaths, other negative consequences for patients and their families), and information about negative impacts of the COVID-19 pandemic on the economy (eg, bankruptcy of businesses, layoffs, pay cuts).

A single item assessed the frequency of thoughtful consideration of the veracity of health information: “When you viewed information about COVID-19, how often did you carefully think about its veracity?” (0=almost never to 3=always). This variable was treated as a moderator.

***Mental Health***

Depressive symptoms were measured using the validated Chinese version of the 9-item Patient Health Questionnaire (PHQ-9) [35]. Participants were asked to rate the frequency at which they experienced each of nine depressive symptoms over the past two weeks (0=never to 3=nearly every day). A mean score was calculated and used for analysis (alpha reliability=.91). A summative score ≥10 indicated the presence of probable moderate to severe depression [36].

Global sleep quality over a 7-day recall period was measured using a single-item Sleep Quality Scale with a rating range from 0 to 10. A higher score indicated better sleep quality. The original score was then recoded into four categories: 1=poor or terrible (0-3), 2=fair (4-6), 3=good (7-9), and 4=excellent (10) [37]. This instrument has been demonstrated to be a reliable and valid measure without significantly increasing respondents’ survey burden.

#### Self-Reported Compliance With Personal Preventive Measures

Participants were asked to report the frequency at which they wore face masks in the workplace and in other public settings (public places and transportation) in the past month (response categories: every time, often, sometimes, never). A composite variable of “consistent face mask wearing” was created to represent respondents who reported wearing a face mask every time both in the workplace and in other public settings (0=no, 1=yes). Participants also reported the frequency at which they sanitized their hands using soaps, liquid soaps, or alcohol-based hand sanitizers after returning from public spaces or touching public installations and equipment (eg, handrails, escalator control panels, or door knobs) (response categories: every time, often, sometimes, never). This item was also recoded into a binary variable of “sanitizing hands every time” (0=no, 1=yes).

### Statistical Analysis

The characteristics of all studied variables were described first. The associations between independent variables (including overall amount, sources, and content of COVID-19–specific information exposure) and mental health outcomes (depressive symptoms and sleep quality) were tested using multivariable linear regression analysis; the associations between the independent variables and behavioral outcomes (self-reported consistent face mask wearing and sanitizing hands every time) were tested using multivariate logistic regression analysis. Background characteristics that were significantly associated with the dependent variables were adjusted for in the regression models. Adjusted unstandardized coefficients (B) or adjusted odds ratios (AORs) and their 95% CIs were reported. An interaction term was further created by multiplying overall information exposure and thoughtful consideration of information veracity, and this term was included in the multivariable linear and logistic regression models to test its significance. SPSS version 24 (IBM Corporation) was used to conduct all analyses, with a two-sided *P* value <.05 indicating statistical significance.

## Results

### Background Characteristics

Over half of the 3035 participants were <30 years of age (1552, 51.1%), male (1612, 53.1%), married (1812, 59.7%), had attained an education level lower than college or university (2004, 66%), had a monthly income level lower than ¥5000 (US $714) (1542, 50.8%), were frontline workers (1847, 60.9%), and were working in an electronic device manufacturing factory (2353, 77.5%) (Table 1).

#### COVID-19–Specific Information Exposure

Of the 3035 participants, 1638 (54.0%), 1057 (34.8%), and 1013 (33.3%) had been exposed to COVID-19–specific information via official web-based media, unofficial web-based media, and television for >1 hour per day, respectively. Smaller proportions reported a daily exposure of >1 hour via newspaper (499/3035, 16.5%) and face-to-face communication (506/3035, 16.7%). Regarding the contents of the information, over half of the 3035 participants were always exposed to statistics about the COVID-19 pandemic (1720, 56.7%), positive information about governmental responses to COVID-19 (1617, 53.3%), and heroic stories about frontline health care workers (1542, 50.8%). Only 1065 of the 3035 participants (35.1%) always thought carefully about the veracity of the COVID-19–specific information (Table 2).

**Table 1:** Background characteristics of the study sample (N=3035), n (%).

Characteristic			Value
**Age (years)**			
	18-25	653 (21.5)	
	26-30	899 (29.6)	
	31-40	1195 (39.4)	
	>40	288 (9.5)	
**Gender**			
	Male	1612 (53.1)	
	Female	1423 (46.9)	
**Marital status**			
	Unmarried	1223 (40.3)	
	Married	1812 (59.7)	
**Highest education level attained**			
	Junior high or below	1163 (38.3)	
	Senior high or equivalent	841 (27.7)	
	College or university	895 (29.5)	
	Postgraduate	136 (4.5)	
**Monthly personal income (¥)^a^**			
	<3000	179 (5.9)	
	3000-4999	1363 (44.9)	
	5000-6999	763 (25.1)	
	7000-9999	327 (10.8)	
	≥10,000	403 (13.3)	
**Type of work**			
	Frontline worker	1847 (60.9)	
	Manager	1188 (39.1)	
**Factory type**			
	Electronic device manufacturing	2353 (77.5)	
	Watchmaking	307 (10.1)	
	Beverage manufacturing	191 (6.3)	
	Biotechnology product manufacturing	184 (6.1)	

^a^1 ¥=US $0.14 on March 1, 2020.

**Table 2:** Exposure to COVID-19–specific information in the study sample (N=3035).

Characteristic			Value	
**Daily average hours of exposure to COVID-19–specific information through different channels,** **n (%)**				
	**Official web-based media**			
		Almost never		134 (4.4)
		Less than 1 hour		1263 (41.6)
		1-2 hours		911 (30.0)
		3-4 hours		258 (8.5)
		More than 4 hours		469 (15.5)
	**Unofficial web-based media**			
		Almost never		543 (17.9)
		Less than 1 hour		1435 (47.3)
		1-2 hours		572 (18.8)
		3-4 hours		186 (6.1)
		>4 hours		299 (9.9)
	**Television**			
		Almost never		614 (20.2)
		Less than 1 hour		1408 (46.4)
		1-2 hours		608 (20.0)
		3-4 hours		147 (4.8)
		>4 hours		258 (8.5)
	**Newspapers and magazines**			
		Almost never		1628 (53.6)
		Less than 1 hour		908 (29.9)
		1-2 hours		294 (9.7)
		3-4 hours		78 (2.6)
		>4 hours		127 (4.2)
	**Face-to-face communication**			
		Almost never		1269 (41.9)
		Less than 1 hour		1260 (41.5)
		1-2 hours		309 (10.2)
		3-4 hours		76 (2.5)
		>4 hours		121 (4.0)
Overall Information Exposure Scale (5 items; sum score), mean (SD)			6.26 (3.92)	
**Frequency of exposure to different topics of COVID-19–specific information, n (%)**				
	**Statistics about the COVID-19 pandemic**			
		Almost never		248 (8.2)
		Seldom		318 (10.5)
		Sometimes		749 (24.7)
		Always		1720 (56.7)
	**Positive information about governmental responses to COVID-19**			
		Almost never		155 (5.1)
		Seldom		260 (8.6)
		Sometimes		1003 (33.0)
		Always		1617 (53.3)
	**Negative information about governmental responses to COVID-19**			
		Almost never		290 (9.6)
		Seldom		445 (14.7)
		Sometimes		1138 (37.5)
		Always		1162 (38.3)
	**Information about development of vaccines and effective treatment**			
		Almost never		196 (6.5)
		Seldom		404 (13.3)
		Sometimes		1156 (38.1)
		Always		1279 (42.1)
	**Heroic stories about frontline health care workers**			
		Almost never		145 (4.8)
		Seldom		227 (7.5)
		Sometimes		1121 (36.9)
		Always		1542 (50.8)
	**Positive information about patients with COVID-19**			
		Almost never		155 (5.1)
		Seldom		297 (9.8)
		Sometimes		1174 (38.7)
		Always		1409 (46.4)
	**Negative information about patients with COVID-19**			
		Almost never		357 (11.8)
		Seldom		594 (19.6)
		Sometimes		1190 (39.2)
		Always		894 (29.5)
	**Information about negative impacts of the COVID-19 pandemic on the economy**			
		Almost never		515 (17.0)
		Seldom		651 (21.4)
		Sometimes		1097 (36.1)
		Always		772 (25.4)
**Thoughtful consideration of the veracity of COVID-19–specific information, n (%)**				
	Almost never		207 (6.8)	
	Seldom		346 (11.4)	
	Sometimes		1417 (46.7)	
	Always		1065 (35.1)	

**Mental Health and Self-Reported Compliance With Personal Preventive Measures**

The prevalence of probable moderate to severe depression was 170/3035 (5.6%), and that of good or excellent sleep quality was 2110/3035 (69.5%) (Table 3). Regarding behavioral responses, 2903/3035 (95.7%) and 2151/3035 (70.9%) participants reported consistent face mask wearing in any public places and sanitizing hands every time after returning from public spaces or touching installations, respectively.

**Table 3:** Mental health and behavioral outcomes in the study sample (N=3035).

Characteristic			Value
**Mental health outcomes**			
	Depressive symptoms (mean score of the PHQ-9^a^), mean (SD)		2.12 (4.02)
	**Sleep quality**		
		Poor or terrible, n (%)	112 (3.7)
		Fair, n (%)	813 (26.8)
		Good, n (%)	1384 (45.6)
		Excellent, n (%)	726 (23.9)
		Mean score (SD)	2.90 (0.80)
**Self-reported compliance with personal preventive measures, n (%)**			
	**Self-reported consistent face mask wearing (wearing a face mask every time in the workplace and in other public spaces or transportation)**		
		No	132 (4.3)
		Yes	2903 (95.7)
	**Self-reported hand sanitizing every time after returning from public spaces or touching installations**		
		No	884 (29.1)
		Yes	2151 (70.9)

^a^PHQ-9: 9-item Patient Health Questionnaire.

### Associations Between Information Exposure and Mental Health Outcomes

In the univariate analysis, age, marital status, education level, monthly personal income, status as frontline worker or manager, and factory type were significantly associated with depressive symptoms and sleep quality (Multimedia Appendix 1).

After adjusting for these significant background characteristics, a higher overall amount of COVID-19–specific information exposure was associated with higher depressive symptoms (adjusted B=0.05, *P*=.006). Specifically, higher exposure via unofficial web-based media (adjusted B=0.20, *P*=.001) and face-to-face communication (adjusted B=0.45, *P*<.001) was associated with higher depressive symptoms. Exposure to negative information about patients with COVID-19 (adjusted B=0.15, *P*=.049) and about effects on the economy (adjusted B=0.31, *P*<.001) was associated with higher depressive symptoms, while exposure to information about development of vaccines and effective treatments was associated with lower depressive symptoms (adjusted B –0.16, *P*=.045) (Table 4).

A higher overall amount of COVID-19–specific information exposure was associated with better sleep quality (adjusted B=0.01, *P*=.01). Specifically, exposure via face-to-face communication was associated with poorer sleep quality (adjusted B=–0.04, *P*=.01), while exposure via television (adjusted B 0.05, *P*<.001) and newspapers and magazines (adjusted B=0.07, *P*<.001) was associated with better sleep quality. Exposure to negative information about patients with COVID-19 (adjusted B=–0.03, *P*=.04) and negative impacts on the economy (adjusted B=–0.04, *P*=.02) was associated with poorer sleep quality. In contrast, exposure to heroic stories about frontline health care workers (adjusted B=0.06, *P*=.002) and positive information about patients with COVID-19 (adjusted B=0.04, *P*=.02) was associated with better sleep quality.

**Table 4:** Linear regression on exposure to COVID-19–specific information and mental health outcomes (N=3035).

Exposure		Depressive symptoms		Sleep quality	
		Adjusted B (95% CI)	*P* value	Adjusted B (95% CI)	*P* value
**Frequency of exposure to COVID-19–specific information through different channels**					
	Official web-based media	0.10 (–0.02 to 0.23)	.11	0.01 (–0.01 to 0.04)	.30
	Unofficial web-based media	0.20 (0.08 to 0.33)	.001	0.02 (–0.01 to 0.04)	.20
	Television	–0.02 (–0.15 to 0.11)	.76	0.05 (0.03 to 0.08)	<.001
	Newspapers and magazines	–0.01 (–0.15 to 0.13)	.87	0.07 (0.04 to 0.10)	<.001
	Face-to-face communication	0.45 (0.30 to 0.60)	<.001	–0.04 (–0.07 to –0.01)	.01
	Overall information exposure	0.05 (0.02 to 0.09)	.006	0.01 (0.00 to 0.02)	.01
**Frequency of exposure to different contents of COVID-19–specific information**					
	Statistics about the COVID-19 pandemic	0.02 (–0.13 to 0.18)	.76	–0.02 (–0.05 to 0.01)	.15
	Positive information about governmental responses to COVID-19	–0.13 (–0.30 to 0.04)	.12	0.02 (–0.01 to 0.06)	.15
	Negative information about governmental responses to COVID-19	0.01 (–0.14 to 0.16)	.86	0.00 (–0.03 to 0.03)	.89
	Information about development of vaccines and effective treatment	–0.16 (–0.32 to –0.00)	.045	0.03 (–0.00 to 0.06)	.08
	Heroic stories about frontline health care workers	–0.13 (–0.30 to 0.05)	.16	0.06 (0.02 to 0.09)	.002
	Positive information about patients with COVID-19	–0.11 (–0.28 to 0.06)	.21	0.04 (0.01 to 0.08)	.02
	Negative information about patients with COVID-19	0.15 (0.00 to 0.29)	0.049	–0.03 (–0.06 to –0.00)	.04
	Information about negative impacts of the COVID-19 pandemic on the economy	0.32 (0.18 to 0.46)	<.001	–0.04 (–0.06 to –0.01)	.02

### Associations Between Information Exposure and Behavioral Outcomes

In the univariate analysis, age, gender, marital status, education level, and factory type were associated with face mask wearing and sanitizing hands every time (Multimedia Appendix 1).

After adjusting for these significant background characteristics, the overall or source-specific amount of COVID-19–specific information exposure was not significantly associated with consistent face mask wearing. Exposure to statistics about the COVID-19 pandemic (AOR 1.23, 95% CI 1.04-1.45; *P*=.02), negative information about governmental responses (AOR 1.32, 95% CI 1.11-1.56; *P*=.001), heroic stories about frontline health care workers (AOR 1.30, 95% CI 1.08-1.56; *P*=.007), and positive information about patients with COVID-19 (AOR 1.33, 95% CI 1.11-1.60; *P*=.002) were positively associated with this outcome.

Overall amount of COVID-19–specific information exposure (AOR=1.03, 95% CI 1.01-1.05; *P*=.003) and specific exposure through official web-based media, unofficial web-based media, television, and newspapers and magazines, with AORs ranging from 1.07 (95% CI 1.00-1.15) to 1.21 (95% CI 1.11-1.32), were positively associated with sanitizing hands every time, while a negative association was found for exposure through face-to-face communication (AOR=0.91, 95% CI 0.84-0.98; *P*=.02). Exposure to all eight information topics was positively associated with this outcome, with AORs ranging from 1.09 (95% CI 1.01-1.18) to 1.36 (95% CI 1.24-1.49) (Table 5).

**Table 5:** Logistic regression of media exposure to COVID-19–specific information and behavioral outcomes (N=3035).

Media exposure		Consistent face mask wearing		Sanitizing hands every time	
		Adjusted odds ratio (95% CI)	*P* value	Adjusted odds ratio (95% CI)	*P* value
**Frequency of exposure to COVID-19–specific information through different channels**					
	Official web-based media	1.01 (0.87-1.18)	.90	1.07 (1.00-1.15)	.05
	Unofficial web-based media	1.03 (0.88-1.20)	.74	1.08 (1.00-1.16)	.04
	Television	1.11 (0.94-1.30)	.22	1.18 (1.09-1.27)	<.001
	Newspapers and magazines	1.03 (0.87-1.23)	.70	1.21 (1.11-1.32)	<.001
	Face-to-face communication	1.13 (0.92-1.37)	.24	0.91 (0.84-0.98)	.02
	Overall information exposure	1.02 (0.97-1.07)	.39	1.03 (1.01-1.05)	.003
**Frequency of exposure to different COVID-19–specific information**					
	Statistics about the COVID-19 pandemic	1.23 (1.04-1.45)	.02	1.23 (1.13-1.33)	<.001
	Positive information about governmental responses to COVID-19	1.16 (0.96-1.40)	.12	1.34 (1.22-1.47)	<.001
	Negative information about governmental responses to COVID-19	1.32 (1.11-1.56)	.001	1.20 (1.10-1.30)	<.001
	Information about development of vaccines and effective treatment	1.16 (0.97-1.39)	.11	1.36 (1.25-1.49)	<.001
	Heroic stories about frontline health care workers	1.30 (1.08-1.56)	.007	1.32 (1.20-1.46)	<.001
	Positive information about patients with COVID-19	1.33 (1.11-1.60)	.002	1.36 (1.24-1.49)	<.001
	Negative information about patients with COVID-19	1.17 (0.98-1.38)	.08	1.14 (1.05-1.23)	.002
	Information about negative impacts of the COVID-19 pandemic on the economy	1.13 (0.95-1.35)	.16	1.09 (1.01-1.18)	.03

### Moderation Effects of Thoughtful Consideration of Information Veracity

**Figure 2:**
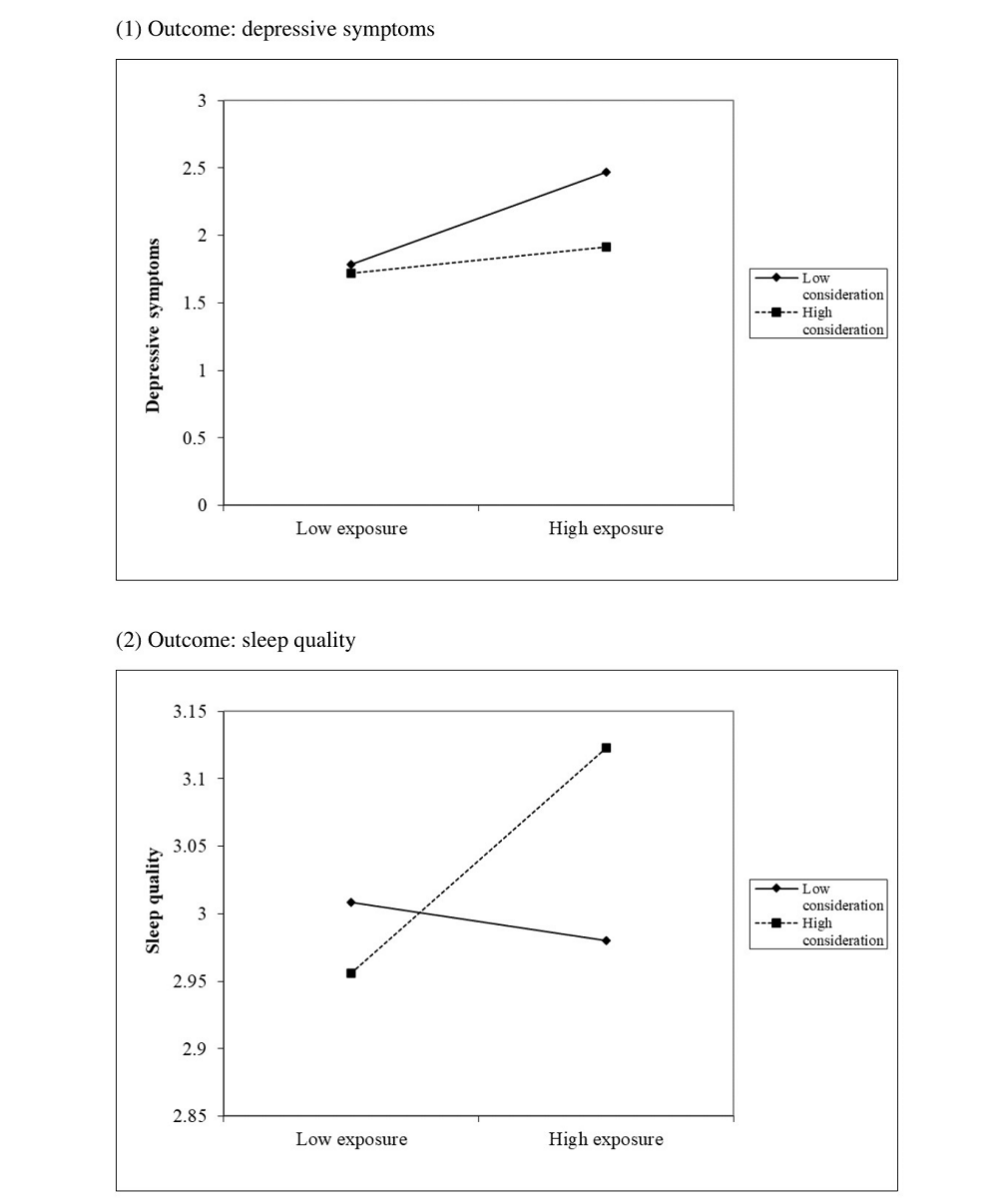
The moderation effects of thoughtful consideration about the veracity of COVID-19–specific information on associations between overall information exposure and the mental health outcomes of (1) depressive symptoms and (2) sleep quality with adjustment for significant background factors.

The interaction term was significantly associated with depressive symptoms (adjusted B=–0.04, *P*=.047) and sleep quality (adjusted B=0.01, *P*<.001). Overall amount of information exposure was associated with higher depressive symptoms among participants who were less likely to consider the veracity of the information to which they were exposed; this association was not significant among those who were more likely to consider the veracity of this information. In contrast, overall amount of information exposure was associated with better sleep quality among participants who were more likely to consider the veracity of the information to which they were exposed; this association was not significant among those who were less likely to consider the veracity of this information (Figure 2 and Multimedia Appendix 2). Thoughtful consideration of information veracity did not moderate the association between overall amount of information exposure and behavioral responses.

## Discussion

### Principal Findings

The COVID-19 pandemic may have increased mental health problems among Chinese factory workers after work resumption, as their prevalence of moderate to severe depression was higher than that observed in the general population before the pandemic (5.6% versus 2.4%) [38]. The prevalence of terrible or poor sleep quality was lower than that of Chinese workers who returned to work in Jiangsu Province (14.9%) [39]. Regarding self-reported compliance with personal preventive measures, the prevalence of consistent face mask wearing in all public spaces was very high, which is crucial for COVID-19 prevention in factories where physical distancing cannot be guaranteed. Despite the WHO recommendation on hand hygiene [7], few participants always sanitized their hands. The importance of hand hygiene must be emphasized during work resumption.

Our findings suggest that the COVID-19 pandemic triggered intensive media responses, as our participants had been exposed to large amounts of COVID-19–specific information every day following the outbreak in China. Web-based media and television were the most common information sources, while newspapers and magazines and face-to-face communication were less common. These findings are consistent with previous reports, which showed a substantial increase in the use of web-based media and television following the COVID-19 outbreak [40].

Consistent with previous studies [4,24], we found that different sources of information exposure had differing effects on mental health and behavioral outcomes. First, television, newspapers, and magazines are traditional media outlets that are mainly operated by governmental organizations in China. Exposure to COVID-19–specific information through these channels not only increased compliance with personal preventive measures but also improved mental health and well-being. These media channels mainly report information verified by expert sources [4,24]. Previous studies suggested that the more people read newspapers and watched television reports about MERS, the more knowledge they acquired about the disease and its prevention strategies [4,24]. Being knowledgeable about COVID-19 was associated with better compliance with personal preventive measures [21] and lower levels of worry or panic [41]. In addition to knowledge dissemination, these traditional media outlets in China often disseminate information about the government’s preparedness plans, actions, and progress in controlling COVID-19. This information is helpful to build trust in the government’s capacity to counter the pandemic. Trust in government was positively associated with adoption of personal preventive measures and mental health during an infectious disease pandemic [42,43].

It was interesting to find that information exposure through official web-based media was associated with better hand hygiene but did not affect mental health status. In China, these media outlets are operated by governmental organizations and disseminate similar information to traditional media. However, instead of one-way communication, official web-based media platforms are interactive and allow readers to leave comments. We speculate that people viewed both positive and negative feedback for specific topics, which may have offset the positive influence on mental health. Information exposure through unofficial web-based media sources was likely to have both positive and negative aspects, as it motivated personal preventive measures but triggered mental health problems at the same time. These findings are similar to studies conducted during the 2015 MERS outbreak in South Korea [4,24]. Compared to official media sources (official web-based media and traditional media in China), unofficial web-based media sources contained not only factual information but also emotional content [22,44]. Negative emotions and unnecessary sensationalism are more likely to be present and amplified in these media outlets during an infectious disease outbreak than in official media channels.

Information exposure through face-to-face communication was limited, probably due to strict control measures implemented by the Chinese government to curb the COVID-19 pandemic (eg, intracity travel restrictions, community lockdown). However, the influence of face-to-face communication cannot be neglected. Higher levels of information exposure through this source were associated with decreased levels of hand hygiene and poorer mental health. It is likely that false and unverified information was disseminated by face-to-face communication during the pandemic. Studies have shown that “bottom-up” misinformation accounted for a significant proportion of information sharing between laypersons during the COVID-19 pandemic [45]. The consequences of misinformation can be long-lasting and should not be underestimated in health crisis management [29].

Our study covered eight types of COVID-19–related content that were active topics attracting public attention from the Chinese population during the pandemic [44,46]. Exposure to all these types of content was associated with increased compliance with face mask wearing and/or hand hygiene. A sufficient amount of information exposure about COVID-19, regardless of topic and valence (positive, negative, or neutral), could potentially cultivate a global sense of emergency and enhance compliance with these preventive measures. Exposure to content about positive outcomes for patients with COVID-19, development of effective treatments and vaccines, and heroic stories about frontline health care workers were associated with better mental health. This is understandable, as these types of information can increase people’s confidence in the control of the pandemic and hence reduce their concern. Moreover, heroic stories may increase altruism, which has a protective effect on mental health during a pandemic. In contrast, negative information about patients with COVID-19 and about impacts on the economy were associated with decreased mental health. Observing the detrimental consequences of COVID-19 may induce fear, according to fear appeal theory [47]. Pay cuts or layoffs resulting from COVID-19 are potential stressors that may lead to worse mental health [48].

The measure of thoughtful consideration inherently assessed the participants’ motivation to scrutinize information quality but also the ability to acquire, discern, and understand accurate health information [49-51]. During the global epidemic of misinformation that has accompanied the COVID-19 pandemic, some individuals were able to actively protect themselves against the crisis. Our results highlighted that thoughtful consideration of the veracity of information to which the participants were exposed significantly moderated the association between overall amount of information exposure and mental health. Participants who thought more about information veracity suffered less from the adverse effect of high information exposure on depression and even had better sleep quality compared to people with low information exposure. People with inadequate skills and resources to analyze and appraise the information to which they are exposed may be particularly vulnerable to mental distress due to high information anxiety, confusion about information quality, and unsatisfied information needs [12,52,53]. There is a significant need for intervention, as only 35% of the participants always thought carefully about the veracity of COVID-19–specific information.

### Implications

Our findings may inform strategic risk communication by government and public health authorities during the COVID-19 outbreak and future public health emergencies to build public trust and facilitate prevention without amplifying panic. Future risk communication related to COVID-19 in China should make use of official web-based media and traditional media platforms; increase dissemination of information about patient rehabilitation, development of effective treatments and vaccines, and heroic deeds of frontline health care workers; and inform the public about effective strategies to reactivate the economy. Pilot testing of communication campaign messages among the target audience is also recommended. Furthermore, it is imperative to invest in building communication capacity to combat the crisis of misinformation. Government and public health authorities should proactively identify misinformation, provide clarifications, and empower the public with adequate skills to critically evaluate the veracity of information.

### Limitations

The present study has some limitations. First, causal relationships between information exposure and the studied outcomes cannot be determined due to the cross-sectional design. Reverse relationships are possible; for example, individuals with mental distress may spend more time searching for COVID-19–related information [16]. Second, the survey did not capture the temporal pattern of information exposure and its correlations with health outcomes [46]; instead, global indicators were used to measure the average exposure levels over a critical period from the initial outbreak to the containment phase. Third, the measures did not differentiate active and passive information gathering; however, the majority of the Chinese population proactively sought related information during the COVID-19 pandemic [21,40]. Fourth, generalization of the evidence from a workforce sample should be made cautiously. The mental health impact of information exposure may be different for people who are unemployed or retired. This study was conducted in one city; however, the participants had resided in 29 of the 34 provincial regions in China prior to work resumption, and the observed findings are thus not only limited to the context of Shenzhen. Moreover, the data were self-reported and verification was not feasible. Participants may also overreport their compliance with personal preventive measures due to social desirability. The web-based survey was anonymous and did not collect participants’ personal details. These measures may have reduced the degree of social desirability bias.

### Conclusion

This study provides empirical evidence of how different dimensions of COVID-19–specific information exposure influenced mental health status and compliance with personal preventive measures at the initial phase of work resumption in China. Thoughtful consideration of the veracity of information played an important role in moderating the associations between information exposure and mental health. These findings can help improve crisis communication during the response to the ongoing pandemic.

## Appendix

Multimedia Appendix 1 Associations between background factors and behavioral/mental health outcomes (N=3035).

Multimedia Appendix 2 Regression coefficients for moderation analyses.

## Abbreviations

AOR: adjusted odds ratio
MERS: Middle East respiratory syndrome
SARS: severe acute respiratory syndrome
WHO: World Health Organization
