# Supplementary material for: Associations of Mental Health and Personal Preventive Measure Compliance With Exposure to COVID-19 Information During Work Resumption Following the COVID-19 Outbreak in China: Cross-Sectional Survey Study
Source: J Med Internet Res. 2020 Oct 8;22(10):e22596. doi: 10.2196/22596 (PMC7546870; doi:10.2196/22596)
Supplement: Multimedia Appendix 1 [file jmir_v22i10e22596_app1.docx]

Multimedia appendix 1. Associations between background factors and behavioral/mental health outcomes (N=3,035).

|  | **Consistent face mask wearing** | **Sanitizing hands every time** | **Depressive symptoms** | **Sleep quality** |
| --- | --- | --- | --- | --- |
|  | **Odds Ratio (95%CI)** | | **B (95%CI)** | |
| Age (years) | 0.83 (0.68, 1.00) † | 1.22 (1.12, 1.33) *** | -0.53 (-0.68, -0.37) *** | 0.08 (0.04, 0.11) *** |
| Gender  (Ref category: Male) | 0.82 (0.58, 1.17) | 1.33 (1.14, 1.56) *** | -0.17 (-0.46, 0.12) | 0.04 (-0.02, 0.10) |
| Marital status  (Ref category: unmarried) | 0.99 (0.70, 1.42) | 1.50 (1.28, 1.76) *** | -0.88 (-1.17, -0.59) *** | 0.15 (0.09, 0.21) *** |
| Highest education level attained | 1.51 (1.23, 1.86) *** | 0.90 (0.83, 0.98) * | 0.60 (0.45, 0.75) *** | -0.15 (-0.18, -0.12) *** |
| Monthly personal income (RMB) | 1.35 (1.14, 1.61) *** | 0.97 (0.90, 1.04) | 0.16 (0.03, 0.28)  * | -0.07 (-0.09, -0.04) *** |
| Type of work  (Ref category: frontline workers) | 1.62 (1.10, 2.38) * | 0.99 (0.84, 1.16) | 0.64 (0.35, 0.93) *** | -0.15 (-0.21, -0.09) *** |
| Type of factory ^a^ |  |  |  |  |
| dummy variable 1 | 2.06 (0.90, 4.76) † | 1.94 (1.44, 2.61) *** | -0.14 (-0.62, 0.33) | 0.01 (-0.08, 0.11) |
| dummy variable 2 | 0.45 (0.26, 0.78)  ** | 0.94 (0.69, 1.29) | 0.16 (-0.44, 0.75) | -0.07 (-0.19, 0.50) |
| dummy variable 3 | 0.40 (0.24, 0.69)  ** | 2.14 (1.45, 3.17) *** | 0.94 (0.34, 1.54)  ** | -0.25 (-0.37, -0.13) *** |

† .05<*P*<.10, * *P*<.05, ** *P*<.01, *** *P*<.001

a. Three dummy variables were created to represent four types of factory: 1) electronic devices manufacturers (dummy variable 1&2&3=0); 2) watchmaking factories (dummy variable 1=1, dummy variable 2&3=0); 3) beverages manufacturers (dummy variable 2=1, dummy variable 1&3=0); and 4) biotechnology products manufacturers (dummy variable 3=1, dummy variable 1&2=0).
