# Supplementary material for: Associations of Mental Health and Personal Preventive Measure Compliance With Exposure to COVID-19 Information During Work Resumption Following the COVID-19 Outbreak in China: Cross-Sectional Survey Study
Source: J Med Internet Res. 2020 Oct 8;22(10):e22596. doi: 10.2196/22596 (PMC7546870; doi:10.2196/22596)
Supplement: Multimedia Appendix 2 [file jmir_v22i10e22596_app2.docx]

|  | **Depressive symptoms** | **Sleep quality** | **Consistent face mask wearing** | **Sanitizing hands every time** |
| --- | --- | --- | --- | --- |
|  | **Adjusted B (95%CI)** | | **Adjusted Log (odds ratio) (95%CI)** | |
| Overall information exposure | 0.13 (0.05, 0.22) ** | -0.02 (-0.04, -0.00) * | 0.01 (-0.08, 0.10) | 0.00 (-0.04, 0.05) |
| Thoughtful consideration of information veracity | 0.05 (-0.22, 0.32) | -0.06 (-0.12, -0.01) * | 0.16 (-0.15, 0.47) | 0.05 (-0.10, 0.20) |
| Interaction term | -0.04 (-0.07, -0.00) * | 0.01 (0.01, 0.02) *** | 0.00 (-0.04, 0.04) | 0.01 (-0.01, 0.03) |

Multimedia appendix 2. Regression coefficients for moderation analyses.

* *P*<.05, ** *P*<.01, *** *P*<.001
